# Comparative Analysis of Human Genes Frequently and Occasionally Regulated by m^6^A Modification

**DOI:** 10.1016/j.gpb.2018.01.001

**Published:** 2018-05-03

**Authors:** Yuan Zhou, Qinghua Cui

**Affiliations:** Department of Biomedical Informatics, School of Basic Medical Sciences, Peking University, Beijing 100191, China

**Keywords:** m^6^A, Epitranscriptome, Signaling network, Gene expression regulation, Gene importance

## Abstract

The **m^6^A** modification has been implicated as an important epitranscriptomic marker, which plays extensive roles in the regulation of transcript stability, splicing, translation, and localization. Nevertheless, only some genes are repeatedly modified across various conditions and the principle of m^6^A regulation remains elusive. In this study, we performed a systems-level analysis of human genes frequently regulated by m^6^A modification (m^6^Afreq genes) and those occasionally regulated by m^6^A modification (m^6^Aocca genes). Compared to the m^6^Aocca genes, the m^6^Afreq genes exhibit **gene importance**-related features, such as lower dN/dS ratio, higher protein–protein interaction network degree, and reduced tissue expression specificity. **Signaling network** analysis indicates that the m^6^Afreq genes are associated with downstream components of signaling cascades, high-linked signaling adaptors, and specific network motifs like incoherent feed forward loops. Moreover, functional enrichment analysis indicates significant overlaps between the m^6^Afreq genes and genes involved in various layers of gene expression, such as being the microRNA targets and the regulators of RNA processing. Therefore, our findings suggest the potential interplay between m^6^A epitranscriptomic regulation and other gene expression regulatory machineries.

## Introduction

Various types of RNA modifications can change the chemical or structural properties of the nucleotide residues and thus constitute the core mechanism of the epitranscriptomic regulation [1], [2]. *N*^6^-methyladenosine (m^6^A), which is one of the most important and widespread RNA modifications [3], can be recognized as the molecular tag by its reader proteins. Accumulating evidence has shown that m^6^A is associated with several key biological processes. For example, m^6^A modification can be specifically recognized by the YTH domain family reader proteins YTHDF2 and YTHDF1 to regulate the degradation [4] and translation of RNA transcripts [5] respectively. And such regulatory processes can be facilitated by YTHDF3 [6], [7]. Besides, YTH domain containing reader protein YTHDC1 is involved in the regulation of alternative splicing [8], while YTHDC2 enhances translational efficiency [9]. Other regulatory factors like the eukaryotic translational initiation factor 3 (eIF3) could also read m^6^A modification to trigger the translation initiation [10]. As the modification could change the chemical properties of nucleotide residues, m^6^A may also perturb the local structure of RNA, and the altered structures have been shown to facilitate the binding of other proteins like heterogeneous nuclear ribonucleoprotein C (HNRNPC) to their target RNAs [11], [12]. Notably, besides the coding transcriptome, m^6^A has also been suggested to regulate the biogenesis of non-coding RNAs (ncRNAs) like microRNAs (miRNAs) [13].

Establishment of immunoprecipitation-based high-throughput sequencing techniques like MERIP-seq or m^6^A-seq greatly facilitates the transcriptome-wide identification of m^6^A modification sites [14], [15]. Data generated from such studies have been curated in the MeT-DB database [16], [17]. Moreover, the transcriptome-wide m^6^A mapping studies also benefit from the recently developed high-resolution m^6^A mapping technique, like miCLIP [18], and computational m^6^A site prediction tools, like the yeast m^6^A predictor m^6^Apred [19] and the mammalian m^6^A predictor SRAMP [20]. Currently, most of the m^6^A modification profiles have been collected in the RMBase database [21] and the MeT-DB database [16], [17]. Therefore, m^6^A sites constitute the vast majority of the RNA methylation sites in both databases. Although m^6^A profiles from various conditions have been included in these databases, the distribution of m^6^A modified genes across these conditions remains unclear. Interestingly, in our initial efforts to compile a comprehensive m^6^A dataset (see details in Table S1) from the MeT-DB V2.0 [17], we noted that only few genes (18 genes) are always modified across all 38 conditions covered in this dataset. Why are some genes regulated by m^6^A modification more extensively than others are? To address this question, we analyzed differences in the conservation, network, regulation, and functional features between gene frequently regulated by m^6^A (m^6^Afreq genes) and those occasionally regulated by m^6^A (m^6^Aocca genes).

## Results and discussion

### m^6^Afreq genes show gene importance-related features

**Figure 1:**
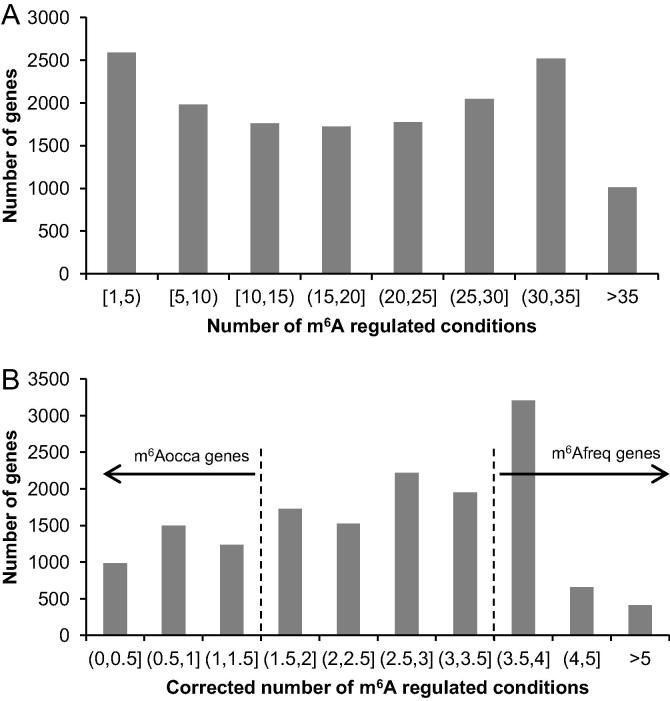
**The overall distribution of the number of m^6^A regulated conditions** **A.** The raw count of the number of m^6^A regulated conditions in our comprehensive m^6^A dataset. Intuitively, an m^6^A regulated condition is counted if there is any m^6^A peak identified in a particular gene under a specified condition. **B.** The corrected number of m^6^A regulated conditions in the comprehensive m^6^A dataset. The corrected number of m^6^A regulated conditions was obtained by dividing the number of m^6^A regulated conditions against that of cell types (covered by m^6^A profiles) where the gene shows baseline expression. A gene is considered to show baseline expression in a cell type, if TPM is greater than 0.5 in the corresponding cell type according to the Human Protein Atlas database. TPM, transcripts per kilobase million.

The overall distribution of the m^6^A modified conditions in our comprehensive m^6^A dataset is shown in Figure 1. Many genes (5854 genes) are m^6^A-modified under ≤19 condition(s) and only some genes (1551 genes) are m^6^A-modified under >35 conditions (Figure 1A). Considering not all genes are expressed under the 38 conditions covered in our dataset, we then corrected the number of m^6^A modified conditions by dividing the number of tissue/cell types in which the gene is expressed. As a result, a similar gene distribution was observed (Figure 1B). Among these genes, 4268 genes are found to be m^6^Afreq genes (modified under >3.5 corrected number of conditions), whereas 3711 genes are found to be m^6^Aocca genes (modified under ≤1.5 corrected number of conditions). To probe the biological characteristics related to such distribution, we performed comprehensive analyses to compare the features of m^6^Afreq genes and m^6^Aocca genes.

Genes expressed across many conditions and cell types tend to be essential genes. Therefore, it is interesting to check whether m^6^Afreq genes possess the essential gene-related features. Although essential genes are often defined in a context-dependent manner, several gene features, including higher conservation, higher protein–protein interaction (PPI) network degree, and broader gene expression spectrum, have been repeatedly shown to be correlated with gene importance [22], [23]. Compared to the m^6^Aocca genes, the m^6^Afreq genes are more conserved as indicated by the significantly lower sequence divergence rate (*i.e.*, lower dN/dS ratio; 0.116 ± 0.00182 *vs*. 0.157 ± 0.00275, Wilcoxon’s test *P* = 7.63E−36), although there are fewer orthologous genes across various species for m^6^Afreq genes (102 ± 2.80 *vs*. 127 ± 4.36, Wilcoxon’s test *P* = 0.0389). Moreover, the m^6^Afreq genes have higher PPI network degree (44.1 ± 1.23 *vs*. 28.0 ± 0.921, Wilcoxon’s test *P* = 8.12E−64), indicating that they tend to interact with more genes and show higher importance in the PPI network. Genes that are constantly expressed across various tissues, *i.e.*, housekeeping genes, likely play essential roles. Compared to the m^6^Aocca genes, the m^6^Afreq genes show significantly lower tissue expression specificity (0.250 ± 0.00156 *vs*. 0.297 ± 0.00236, Wilcoxon’s test *P* = 1.95E−68), indicating that m^6^Afreq genes tend to be more widely expressed across different tissues.

**Figure 2:**
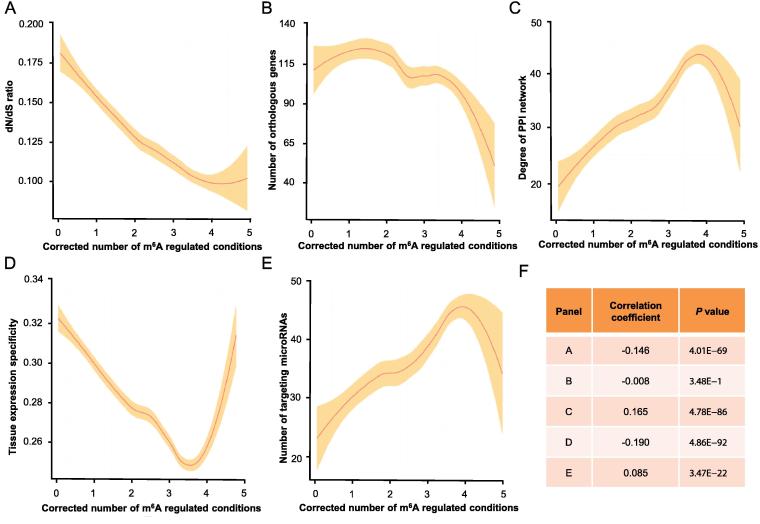
**The correlation between the corrected number of m^6^A regulated conditions and various gene features** The correlation curves between the corrected number of m^6^A regulated conditions and various gene features are plotted by using the LOESS smoothing technique. The line indicates the local average estimated by LOESS smoothing and the shade indicates the confidence interval. Outlier genes (0.5%) with extremely high corrected number of m^6^A regulated conditions are omitted due to their high variation in gene feature values, which could result in badly skewed regression lines. **A.** Correlation of corrected number of m^6^A regulated conditions with dN/dS ratio. **B.** Correlation of corrected number of m^6^A regulated conditions with number of orthologous genes. **C.** Correlation of corrected number of m^6^A regulated conditions with PPI network degree. **D.** Correlation of corrected number of m^6^A regulated conditions with tissue expression specificity. **E.** Correlation of corrected number of m^6^A regulated conditions with number of targeting microRNAs. **F.** The summary of Spearman’s correlation coefficient and *P* values for panels A–E. PPI, protein–protein interaction.

The classification of m^6^Afreq genes and m^6^Aocca genes depends on the threshold used. To avoid bias induced by the arbitrary threshold, we then calculated the Spearman’s correlation coefficients between the corrected number of m^6^A regulated conditions and the aforementioned gene importance-related features. As shown in Figure 2, our results are in line with the m^6^Afreq genes *vs.* m^6^Aocca genes comparisons shown above for most features, with the exception that no significant correlation is observed for the number of orthologous genes. The corrected number of m^6^A regulated conditions shows positive correlations with PPI network degree, but negative correlations with dN/dS ratio and the tissue expression specificity. Given the corrected number of m^6^A regulated conditions is in accordance with most of the aforementioned gene importance-related features (except the number of orthologous genes), genes frequently regulated by m^6^A modification are more likely to be important to the cell.

### Signaling network properties of the m^6^Afreq genes

As shown in the previous section, the m^6^Afreq genes have higher PPI network degree. However, the *in vivo* relationships between genes are more complicated than what is described by the binary PPI network. We thus performed the comprehensive signaling network analysis for the detailed network topology properties of m^6^Afreq genes. Besides PPIs, directed activating (positive) interactions and repressing (negative) interactions between genes are also included in the signaling network.

As a result, 1530 m^6^Afreq genes and 1194 m^6^Aocca genes were mapped onto the signaling network, respectively. No significant difference was observed in the network degree with respect to the directed edges when comparing the m^6^Afreq genes and m^6^Aocca genes (Wilcoxon’s test *P* = 0.611). We tried to classify edges into activating and repressing edges, and compare the degree by considering activating edges or repressing edges alone. We found that compared to m^6^Aocca genes, m^6^Afreq genes have higher network degree when considering repressing edges alone (Wilcoxon’s test *P* = 3.28E−4). More specifically, m^6^Afreq genes have higher negative out-degree (*i.e.*, the number of signal receivers repressed by this gene) than m^6^Aocca genes (1.63 ± 0.116 *vs*. 0.965 ± 0.0729, Wilcoxon’s test *P* = 1.05E−7), indicating that m^6^Afreq genes tend to repress other genes in the signaling network. We also tested other node centrality properties, including betweenness centrality, closeness centrality, eigenvector centrality, and transitivity centrality. Most of these properties do not significantly differ between m^6^Afreq genes and m^6^Aocca genes (Wilcoxon’s test *P* > 0.05), except that the m^6^Afreq genes show marginally higher betweenness centrality (2.52E−4 ± 3.18E − 5 *vs*. 1.78E−4 ± 2.22E−5, Wilcoxon’s test *P* = 0.0203) and closeness centrality (5.64E−3 ± 2.17E−5 *vs*. 5.62E−3 ± 2.41E−5, Wilcoxon’s test *P* = 0.0285) than m^6^Aocca genes. These results suggest that m^6^Afreq genes and m^6^Aocca genes are of largely comparable importance to the signaling network.

**Figure 3:**
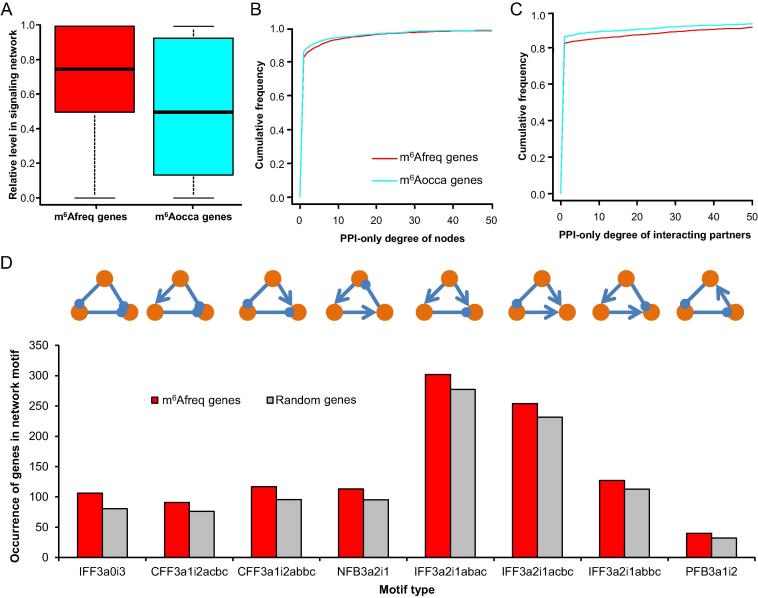
**The network feature of the m^6^Afreq genes** **A.** Boxplot comparing the distributions of relative level in the signaling network, between the m^6^Afreq genes and m^6^Aocca genes. The relative level in the signaling network shown here was calculated as the shortest distance to any upstream receptor divided by the sum of the shortest distance to any upstream receptor and the shortest distance to any downstream transcriptional factors. **B.** Cumulative distribution plot comparing the PPI-only degree distribution of the m^6^Afreq genes and that of m^6^Aocca genes. The PPI-only degree only considers PPI edges in the signaling network but omits the activating and repressing edges. **C.** Cumulative distribution plot comparing the PPI-only degree distribution of the interacting partners of m^6^Afreq genes and that of the interacting partners of m^6^Aocca genes. **D.** The overrepresented network motifs of m^6^Afreq genes. In a network motif, if there are more m^6^Afreq genes than 9500 out of 10,000 sets of randomly picked genes (corresponding to an empirical *P* value < 0.05), this motif is considered as an overrepresented motif. The respective motifs are explicitly depicted by the schemas on top. The activating and repressing edges are indicated using lines with arrowhead and circle, respectively. The name of the motif is composed of the motif type and the code describing the edge topology in the motif. For example, IFF3a1i2abbc indicates an incoherent feedforward loop with three nodes (a, b, and c) that form one activating edge and two repressing edges. Among the three edges, the major class of the edges (in this motif, the major class is repressing edge) comprises the edge between a and b, and the edge between b and c. IFF, incoherent feedforward loop; CFF, coherent feedforward loop; NFB, negative feedback loop; PFB, positive feedback loop.

The difference in betweenness centrality and closeness centrality between m^6^Afreq genes and m^6^Aocca genes also implies that the localization of m^6^Afreq genes and m^6^Aocca genes in the signaling network would differ. To test this hypothesis, for each node, we calculated its shortest distance to the upstream receptors and that to the downstream effectors, and deduced its relative level in the signaling network by comparing these two distances. The relative level of a gene ranges from 0 to 1 with larger values indicative the downstream location (*i.e.*, closer to the downstream effectors than to the upstream receptors) of the gene. While the upstream receptors could be clearly defined by the Gene Ontology (GO) term ‘receptor activity’, the identification of downstream effectors was not straightforward. We adopted two alternative definitions of downstream effectors. First, the downstream effectors could be identified as the nodes with zero out-degree after removing feedback loops. Since no signal would be sent from such kind of nodes, these nodes are intuitively downstream effectors at the bottom ends of signaling cascades. Second, the topology-based definition of downstream effectors could be misled by the incomplete signaling network topology, when the edges in the signaling network are limited. Therefore, we also assigned all transcription factors, which are often the outputting nodes in signaling pathways, as the downstream effectors. When applying topology-based definition of downstream effectors, no difference in signaling network could be observed between m^6^Afreq genes and m^6^Aocca genes (Wilcoxon’s test *P* = 0.289). A more prominent difference was noticed between m^6^Afreq genes and m^6^Aocca genes when we assigned the transcription factors as the downstream effectors (Figure 3A; 0.660 ± 0.00943 *vs*. 0.553 ± 0.0110; Wilcoxon’s test *P* = 2.19E−23). This result indicates that the m^6^Afreq genes, especially transcription factors, tend to act as the downstream effectors along the signaling cascades.

Besides the activating/repressing edges, there are considerable numbers of PPI edges present in the signaling network. Nodes with many PPI partners in the signaling network often act as the adaptors, which can recruit other signaling components for efficient signaling [24]. We checked the PPI-only degree (the degree after ignoring the activating and repressing edges) of m^6^Afreq genes and m^6^Aocca genes. As a result, we found that m^6^Afreq genes have higher PPI-only degree than m^6^Aocca genes (Figure 3B; 2.64 ± 0.161 *vs*. 2.38 ± 0.177, Wilcoxon’s test *P* = 7.27E−5). In addition, the interacting partners of m^6^Afreq genes also exhibited higher PPI-only degree than the partners of m^6^Aocca genes (Figure 3C; 10.2 ± 0.441 *vs*. 8.16 ± 0.443, Wilcoxon’s test *P* = 3.99E−5). Therefore, the m^6^Afreq genes are inclined to be the recruited partners of high-linked signaling adaptors, or they themselves can act as high-linked signaling adaptors.

Signaling cascades are not always linear, and the signaling network motifs like feedback loops and feedforward loops are prevalent to achieve the fine-tuned cellular signaling [25], [26], [27]. We thus tested whether the m^6^Afreq genes were overrepresented in some specific network motifs in comparison with random expectation (see Materials and methods section for details). All overrepresented network motifs are shown in Figure 3D and we found that the m^6^Afreq genes are most overrepresented in various types of incoherent feedforward loops. Unlike the negative feedback loops and coherent feedforward loops, which often work for cellular homeostasis, adaptation, and de-sensitivity, the incoherent feedforward loops are often associated with ultra-sensitivity and non-monotonic response [25], [26], [27], [28]. The m^6^Afreq genes are also overrepresented in specific types of coherent feedforward loops that are unlikely to achieve adaptation [26]. Taken together, these results indicate that the m^6^Afreq genes are more likely to be involved in regulating the signal sensitivity than cellular homeostasis.

### m^6^Afreq genes overlap with microRNA targets and development-related genes

Interestingly, a previous study shows that miRNA targets tend to be the downstream components in the signaling networks, interact with high-linked adaptors, and participate in the positively-linked network motifs [27]. Considering that m^6^Afreq genes show similar network properties, it is interesting to see whether genes extensively regulated by the m^6^A modification are also intensively targeted by miRNAs. We calculated the number of targeting miRNAs on each gene and found that the m^6^Afreq genes are more intensively regulated by miRNAs than the m^6^Aocca genes (number of targeting miRNAs 2.40 ± 0.113 *vs*. 1.18 ± 0.0858; Wilcoxon’s test *P* = 1.23E−20). Moreover, we also observed a positive correlation between the corrected number of m^6^A regulated conditions and the number of targeting miRNAs (Spearman’s correlation = 0.085, *P* = 3.47E−22; Figure 2E). The similar positive correlation persists when the positively co-expressed miRNA–target pairs (which were derived from mirCox database [29], see also Materials and Methods) (Figure S1A; Spearman’s correlation = 0.0562, *P* = 1.43E−10) or the negatively co-expressed miRNA–target pairs were considered alone (Figure S1B; Spearman’s correlation = 0.0514, *P* = 4.42E−9). Together, these results indicate potential crosstalk between m^6^A regulation and miRNA regulation. Recently Molinie et al. have reported that transcript isoforms heavily modified by m^6^A tend to have shorter 3′-UTR and therefore fewer miRNA binding sites [30]. Nevertheless, the conclusions of two studies are not necessarily conflicting: while Molinie et al. focused on the intensively modified RNAs and performed the comparison between transcripts isoforms (*i.e.*, modified isoforms *vs.* non-modified isoforms), in this study we focused on the extensively modified RNAs and performed comparison between different genes (*i.e.*, genes widely modified across various conditions *vs.* genes occasionally modified). It is possible that some genes are surveilled by multiple miRNAs and frequent m^6^A methylation. When heavily methylated, the isoforms lacking miRNA binding sites of such genes could be expressed to escape the regulation of miRNAs; conversely, the isoforms with multiple miRNA binding sites could be expressed when the m^6^A regulation is not present. How the miRNAs and m^6^A cooperate to regulate the gene expression in a sophisticated way deserves further experimental investigation.

miRNAs have been shown to be associated with cell proliferation and apoptosis [31]. We speculate that the m^6^Afreq genes could have similar enriched functions. We thus performed the GO functional enrichment analysis for m^6^Afreq genes. As a result, we found that the m^6^Afreq genes are significantly enriched for the terms like “embryo development”, “mitotic cell cycle”, “growth”, and “apoptotic signaling pathway” (Table S2). It is of note that these terms are not significantly enriched in m^6^Aocca genes (Table S3). This result again indicates potential functional crosstalk between m^6^A modification and miRNA targeting. In addition, m^6^A modification has also been implicated in the regulation of transcript translation, localization, stability, and splicing [4], [5], [8]. Interestingly, m^6^Afreq genes are also significantly associated with the functional terms like “negative regulation of transcription from RNA polymerase II promoter” and “RNA processing” (Table S2), which are not significantly enriched in m^6^Aocca genes (Table S3). Therefore, in addition to directly participating in the RNA metabolism process, it is plausible that m^6^A could also regulate RNA metabolism indirectly via extensively targeting the RNA metabolism-related genes, ultimately achieving more sophisticated regulation of the gene expression.

### Preliminary validation on the quantitative m^6^A dataset and non-methylated genes

In the aforementioned analyses, we focused on the genes that are m^6^A regulated across various conditions. Given these analyses were based only on the binary methylation profiles (*i.e.*, m^6^A modified or not), the m^6^A methylation level was not taken into consideration. Therefore, we also took advantage of the quantitative m^6^A methylation profiles in the MeT-DB V2.0 database [17] for preliminary validation of the main results shown above. These m^6^A methylation profiles were collected using the standardized pipeline, and a quantitative enrichment score was provided for each m^6^A site peak. For each gene, a normalized m^6^A regulation breadth score was calculated in a way similar to the calculation of tissue expression specificity [32], [33] (see also Materials and methods section). The normalized m^6^A regulation breadth ranges from 0 to 1, with higher score indicative genes frequently regulated by m^6^A.

We checked the correlations between the normalized m^6^A regulation breadth and several gene features that have been shown to be associated with m^6^Afreq genes in the analyses above. In line with the results from binary methylation profiles, the normalized m^6^A regulation breadth shows positive correlation with the PPI network degree, the relative level in signaling network, and the number of targeting miRNAs, while a negative correlation of the normalized m^6^A regulation breadth with dN/dS ratio and tissue expression specificity was observed (Figure S2). These results further support our findings from the binary methylation profile analyses.

Another issue of our analyses is that we did not take into consideration the genes that are not methylated. Due to the limited coverage of currently available m^6^A profiles, it is hard to identify *bona fide* non-regulated genes (*i.e.*, m^6^Anone genes) without significant bias. To perform a preliminary test, we defined genes that have baseline expression in at least one cell type covered by the m^6^A profiles but have no known m^6^A sites as the m^6^Anone genes. Consequently, we identified 2779 m^6^Anone genes for comparison of the gene features that have been shown to be associated with m^6^Afreq genes. Generally, the gene features of m^6^Anone genes are much more similar to those of m^6^Aocca genes than to those of m^6^Afreq genes (Figure S3). For example, m^6^Afreq genes have the highest PPI network degree, followed by m^6^Aocca genes, and then m^6^Anone genes. These results are in line with intuitive expectation. We anticipate that with the accumulation of m^6^A profiles in public databases, a less biased comparison between m^6^Afreq genes, m^6^Aocca genes, and m^6^Anone genes will be performed in the future.

Although our analyses suggest largely consistent results about the difference between m^6^Afreq genes and m^6^Aocca genes, substantial limitation exists in this study. First, the current human m^6^A methylation profiles were largely derived from cell lines especially cancer cell lines like HeLa and A549 [16], [17], [21]. Therefore, these profiles could not fully recapitulate the *in vivo* m^6^A methylation patterns in normal human tissues. We hope that more tissue-derived m^6^A profiles can be generated in the future so that a dataset more representative of human biology would be compiled. Second, although we are able to compile a quantitative m^6^A dataset according to the enrichment score of m^6^A methylation peaks, the actual stoichiometry of m^6^A methylation is still hard to be measured using current MeRIP-seq technologies [30], [34]. A novel m^6^A methylation quantification method is crucial to generate less biased methylation profiles for more reliable comparative analyses. Third, it is known that the topology of m^6^A sites along the genes could convey biological functions [14], [15]. However, we did not perform analysis at m^6^A site level in the current study. The recent progress in single-nucleotide m^6^A site mapping technique and m^6^A site prediction methods [18], [20] could enable a comprehensive comparison of m^6^A methylation sites across different conditions. Finally, to study the (functional) conservation of m^6^A modifications, it would also be interesting to evaluate our findings in other species.

In summary, our results indicate that the m^6^A modification tends to regulate important genes. Besides, the miRNA targets and regulators of gene expression like transcriptional factors and RNA processing factors are also suggested to be preferred targets of m^6^A modification. Therefore, extensive functional crosstalk between m^6^A epitranscriptomic regulation and other regulatory machineries of gene expression is implied.

## Materials and methods

### Definition of gene groups based on the number of m^6^A modification conditions

The human m^6^A modification profiles, which cover 38 different m^6^A modification conditions (Table S1), were downloaded from the recently-updated 2.0 version of the MeT-DB database (http://compgenomics.utsa.edu/MeTDB/ and http://www.xjtlu.edu.cn/metdb2) [16]. We first discarded the m^6^A profiles, where the expression of any m^6^A methylation core components (including *METTL3*, *METTL14*, *WTAP*, *ALKBH5*, and *FTO*) was perturbed (knockout, knockdown or, over-expression), and combined the modification sites from the biological replicates. Then, the modification sites were mapped to Entrez genes and the number of conditions when the gene was modified on at least one m^6^A site was counted. To reduce bias, we corrected the number of m^6^A regulated conditions by dividing the number of cell types with baseline expression. For each gene, the number of cell types or tissues covered by m^6^A studies and showing baseline expression (*i.e.*, transcripts per million, TPM >0.5) of this gene was derived from the Human Protein Atlas database (https://www.proteinatlas.org/) [35]. The genes with corrected number of m^6^A regulated conditions >3.5 (roughly corresponding to the top 25% in the distribution of corrected number of m^6^A regulated conditions) were defined as the m^6^Afreq genes, while those with corrected number of m^6^A regulated conditions ≤1.5 (roughly corresponding to the bottom 25% in the distribution) were defined as the m^6^Aocca genes.

We also complied a quantitative m^6^A dataset (m^6^A-quantitative dataset) based on the quantitative methylation profiles from MeT-DB V2.0. Then, the m^6^A peaks were mapped onto the Entrez genes, and the total enrichment score along each transcript was calculated. For the gene with multiple transcripts, only the maximum of the total enrichment scores was retained. The total enrichment score of the genes between different technical replicates were averaged and log_10_-transformed to reduce the bias from the extremely high enrichment scores. Consequently, for each gene in the m^6^A-quantitative dataset, 38 total enrichment scores, which are in correspondence to 38 different conditions, were obtained. Based on these 38 total enrichment scores, a specificity score *τ* is calculated in the same way as the calculation of tissue expression specificity [32], [33]. Finally, the normalized m^6^A regulation breadth was defined as 1 − *τ*. By definition, the normalized m^6^A regulation breadth ranges from 0 to 1, where higher score indicates more frequently regulated genes.

### Statistical analysis of the gene importance-related gene features

The human-to-mouse dN/dS ratios were downloaded from the Ensembl database (http://www.ensembl.org/) [36]. The numbers of orthologous genes were retrieved from the orthologous matrix (OMA) database (http://omabrowser.org/oma/) [37]. The PPI data were obtained from the BioGRID database (http://thebiogrid.org/) [38]. After removing genetic interactions and protein–RNA interactions, the degree of each protein was calculated by counting the total number of its interacting partners [39]. As for the tissue expression specificity, we first obtained the gene expression atlas across 79 human tissues measured by Su et al. [40] (GEO accession number: GDS590). For each gene, the tissue expression specificity was measured according to the state-of-the-art *τ* method which was described in the previous studies [32], [33]. The conversion of gene symbols and RefSeq IDs to Entrez gene ID was performed according to the ID mapping file retrieved from the Ensembl database. All statistical analysis was performed in R (https://www.r-project.org/).

### Signaling network analysis

The most recent human signaling network was downloaded from the Wang lab database (http://www.cancer-systemsbiology.org/) [27]. The node centrality analysis was performed using the igraph package in R. The relative level in the signaling network was calculated as the shortest distance to any upstream receptor divided by the sum of the shortest distance to any upstream receptor and the shortest distance to any downstream effector (*e.g.*, transcriptional factors). Therefore, higher relative level indicates that the gene is located at the downstream of signaling network. The shortest distance between two genes was also calculated using igraph package with the edge direction constraint. The common network motifs in the signaling network were defined in previous work [26]. The total occurrence of one gene in a specific network motif was summarized using an in-house Perl script. We also randomly re-sampled equal number of genes in the signaling network to that of the m^6^Afreq genes or m^6^Aocca genes. This random re-sampling procedure was repeated for 10,000 times, which enables us to evaluate whether the enrichment of m^6^Afreq or m^6^Aocca genes for a specific motif can be also observed in randomly picked genes (thus randomly expected) or not. If the observed real occurrence is higher than the random occurrence for more than 9500 out of 10,000 re-sampling trials, the observed over-representation is considered as non-random (*i.e.*, re-sampling test *P* < 0.05).

### Comparison of microRNA targets and functional association

The experimentally-identified miRNA–target interactions were obtained from the miRTarBase (http://mirtarbase.mbc.nctu.edu.tw/) [41]. To reduce false positive results, only miRNA–target interactions supported by at least one piece of strong evidence record or by at least three pieces of weak evidence records were retained. We also examined the co-expressed miRNA–target database according to the mirCoX database [29]. For each miRNA–gene pair, the mirCoX database calculates the percentiles of correlation coefficients on either miRNA side or gene side. Therefore, the geometric mean of these two percentiles, also known as mutual rank [42], could serve as a reasonable measurement of miRNA–gene co-expression to filter the miRTarBase miRNA–target pairs. We assigned miRNA–target pair that has positive correlation coefficient and mutual rank <0.5 to be the positively co-expressed pairs, and those having negative correlation coefficient and mutual rank >0.5 to be the negatively co-expressed pairs.

The functional enrichment (GO biological process) analysis was performed using gProfileR online tool (http://biit.cs.ut.ee/gprofiler) with default parameters and threshold except the unspecific terms that are associated with more than 1000 genes were excluded before analysis [43]. To reduce the redundant terms, we applied “best per parent group” filtration provided by the gProfileR tool to the significantly-enriched terms.

## Authors’ contributions

YZ and QC conceived and designed the analysis. QC supervised the study. YZ performed the analysis. YZ wrote the manuscript and QC edited the manuscript. Both authors read and approved the final manuscript.

## Competing interests

The authors have declared no competing interests.

## Supplementary material

Supplementary Figure S1 **The correlation between the corrected number of m^6^A regulated conditions and the number of co-expressed targeting microRNAs**The correlation curves between the corrected number of m^6^A regulated conditions and the number of co-expressed targeting microRNAs are plotted using the LOESS smoothing technique. The line indicates the local average estimated by LOESS smoothing and the shade indicates the confidence interval. Outlier genes (0.5%) with extremely high corrected number of m^6^A regulated conditions are omitted due to their high variation in gene feature values, which could result in badly skewed regression lines. **A**. Correlation with the number of positively co-expressed targeting microRNAs. **B**. Correlation with the number of negatively co-expressed targeting microRNAs.

Supplementary Figure S2 **The correlation between the normalized m^6^A regulation breadth and various gene features in the quantitative m^6^A dataset**The correlation curve is plotted by using the LOESS smoothing techniques. The line indicates the local average estimated by LOESS and the shade indicates the confidence interval. **A**. Correlation of normalized m^6^A regulation breadth score with dN/dS ratio. The normalized m^6^A regulation breadth score of one gene summarizes the m^6^A peak scores of the gene across 38 conditions, calculated similarly as for the tissue expression specificity (see Materials & Methods for details). **B**. Correlation of normalized m^6^A regulation breadth score with tissue expression specificity. **C**. Correlation of normalized m^6^A regulation breadth score with PPI network degree. **D**. Correlation of normalized m^6^A regulation breadth score with relative level in signaling network. **E**. Correlation of normalized m^6^A regulation breadth score with number of targeting microRNAs. **F**. The summary of Spearman’s correlation coefficient and *P* values corresponding to the panels A−E. PPI, protein–protein interaction.

Supplementary Figure S3 **Boxplots for the comparison of various gene features between m^6^Anone genes, m^6^Aocca genes, and m^6^Afreq genesA**. Comparison of dN/dS ratio. **B**. Comparison of tissue expression specificity. **C**. Comparison of PPI network degree. Eight proteins with extremely high degree (>800) are considered as outliners and thus not shown in the plot. **D**. Comparison of number of targeting microRNAs. **E**. Comparison of the relative level in the signaling network. **F**. The summary of Wilcoxon’s test *P* values corresponding to the panels A−E.

Supplementary Table S1 **Information about the 38 conditions covered by the comprehensive m^6^A dataset**.

Supplementary Table S2 **Top 20 enriched functional terms for m^6^Afreq genes**.

Supplementary Table S3 **Top 20 enriched functional terms for m^6^Aocca genes**.
