# Supplementary material for: Comparative Analysis of Human Genes Frequently and Occasionally Regulated by m6A Modification
Source: Genomics Proteomics Bioinformatics. 2018 May 3;16(2):127–35. doi: 10.1016/j.gpb.2018.01.001 (PMC6112303; doi:10.1016/j.gpb.2018.01.001)
Supplement: Supplementary Figure S1 — The correlation between the corrected number of m6A regulated conditions and the number of co-expressed targeting microRNAsThe correlation curves between the corrected number of m6A regulated conditions and the number of co-expressed targeting microRNAs are plotted using the LOESS smoothing technique. The line indicates the local average estimated by LOESS smoothing and the shade indicates the confidence interval. Outlier genes (0.5%) with extremely high corrected number of m6A regulated conditions are omitted due to their high variation in gene feature values, which could result in badly skewed regression lines. A. Correlation with the number of positively co-expressed targeting microRNAs. B. Correlation with the number of negatively co-expressed targeting microRNAs. [file mmc1.pptx]

## Slide 1
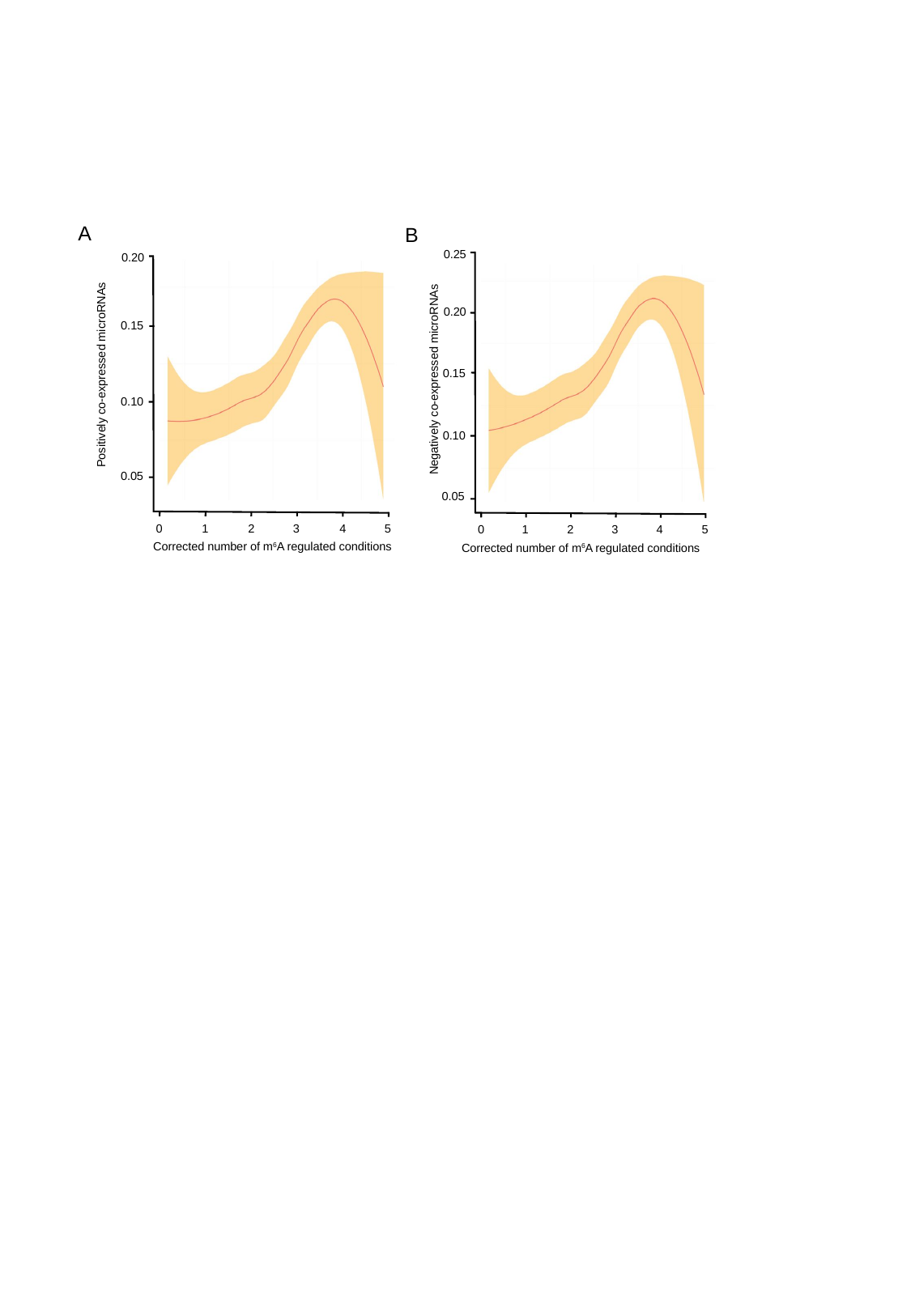

A
B
0.20
0.15
Positively co-expressed microRNAs
0.10
0.05
0
1
2
3
4
5
Corrected number of m6A regulated conditions
0.25
0.20
0.15
Negatively co-expressed microRNAs
0.10
0.05
0
1
2
3
4
5
Corrected number of m6A regulated conditions
