# Supplementary material for: Comparative Analysis of Human Genes Frequently and Occasionally Regulated by m6A Modification
Source: Genomics Proteomics Bioinformatics. 2018 May 3;16(2):127–35. doi: 10.1016/j.gpb.2018.01.001 (PMC6112303; doi:10.1016/j.gpb.2018.01.001)
Supplement: Supplementary Figure S2 — The correlation between the normalized m6A regulation breadth and various gene features in the quantitative m6A datasetThe correlation curve is plotted by using the LOESS smoothing techniques. The line indicates the local average estimated by LOESS and the shade indicates the confidence interval. A. Correlation of normalized m6A regulation breadth score with dN/dS ratio. The normalized m6A regulation breadth score of one gene summarizes the m6A peak scores of the gene across 38 conditions, calculated similarly as for the tissue expression specificity (see Materials & Methods for details). B. Correlation of normalized m6A regulation breadth score with tissue expression specificity. C. Correlation of normalized m6A regulation breadth score with PPI network degree. D. Correlation of normalized m6A regulation breadth score with relative level in signaling network. E. Correlation of normalized m6A regulation breadth score with number of targeting microRNAs. F. The summary of Spearman’s correlation coefficient and P values corresponding to the panels A−E. PPI, protein–protein interaction. [file mmc2.pptx]

## Slide 1
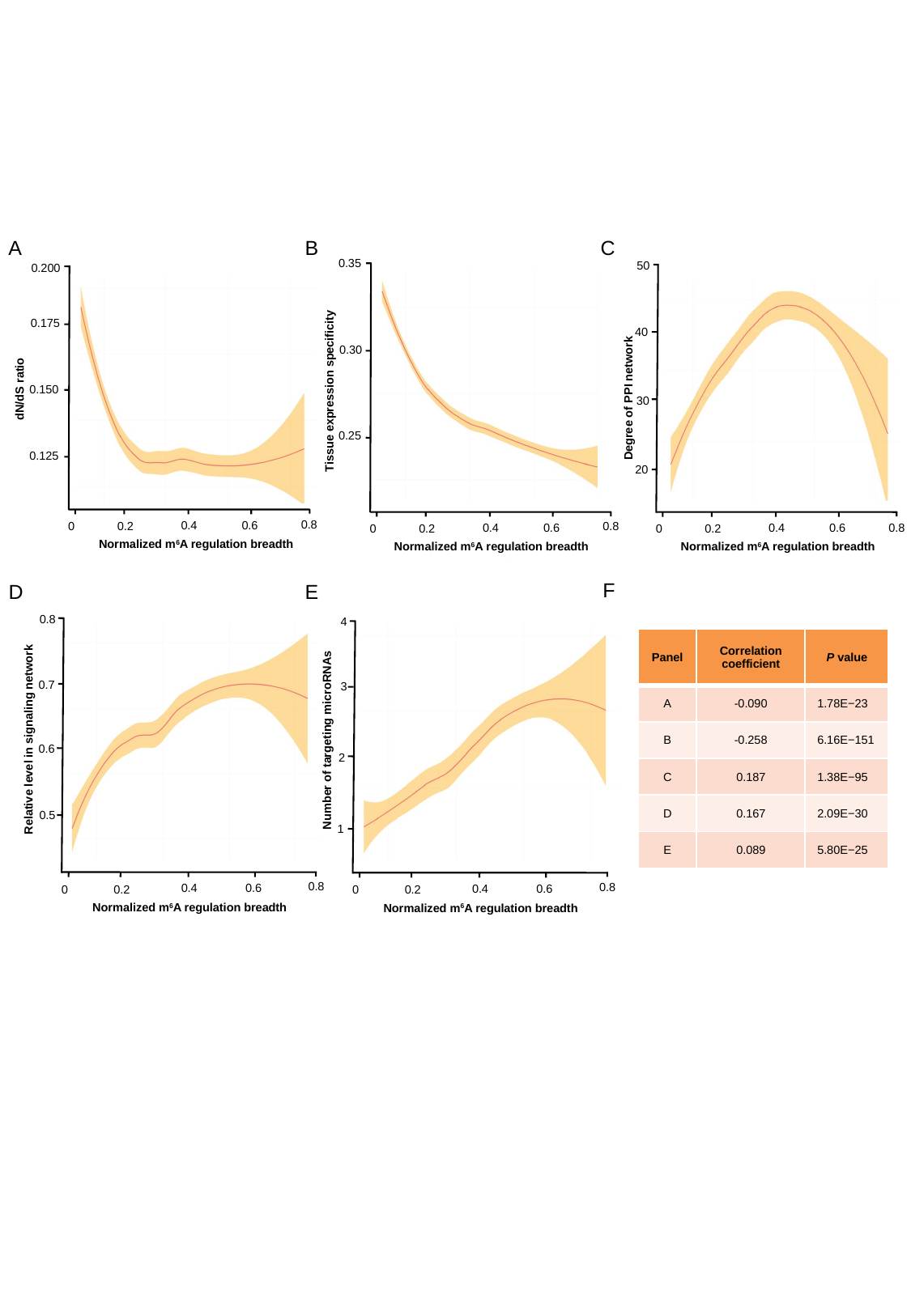

A
B
C
0.200
0.175
0.150
dN/dS ratio
0.125
0.8
0.4
0.6
0
0.2
Normalized m6A regulation breadth
50
40
Degree of PPI network
30
20
0.8
0.4
0.6
0
0.2
Normalized m6A regulation breadth
0.35
 0.30
Tissue expression specificity
 0.25
0.8
0.4
0.6
0
0.2
Normalized m6A regulation breadth
N
N
F
D
E
0.8
0.7
Relative level in signaling network
 0.6
 0.5
0.8
0.4
0.6
0
0.2
Normalized m6A regulation breadth
 4
 3
Number of targeting microRNAs
 2
 1
0.8
0.4
0.6
0
0.2
Normalized m6A regulation breadth
| Panel | Correlation coefficient | P value |
| --- | --- | --- |
| A | -0.090 | 1.78E−23 |
| B | -0.258 | 6.16E−151 |
| C | 0.187 | 1.38E−95 |
| D | 0.167 | 2.09E−30 |
| E | 0.089 | 5.80E−25 |
N
