# Supplementary material for: Comparative Analysis of Human Genes Frequently and Occasionally Regulated by m6A Modification
Source: Genomics Proteomics Bioinformatics. 2018 May 3;16(2):127–35. doi: 10.1016/j.gpb.2018.01.001 (PMC6112303; doi:10.1016/j.gpb.2018.01.001)
Supplement: Supplementary Figure S3 — Boxplots for the comparison of various gene features between m6Anone genes, m6Aocca genes, and m6Afreq genesA. Comparison of dN/dS ratio. B. Comparison of tissue expression specificity. C. Comparison of PPI network degree. Eight proteins with extremely high degree (>800) are considered as outliners and thus not shown in the plot. D. Comparison of number of targeting microRNAs. E. Comparison of the relative level in the signaling network. F. The summary of Wilcoxon’s test P values corresponding to the panels A−E. [file mmc3.pptx]

## Slide 1
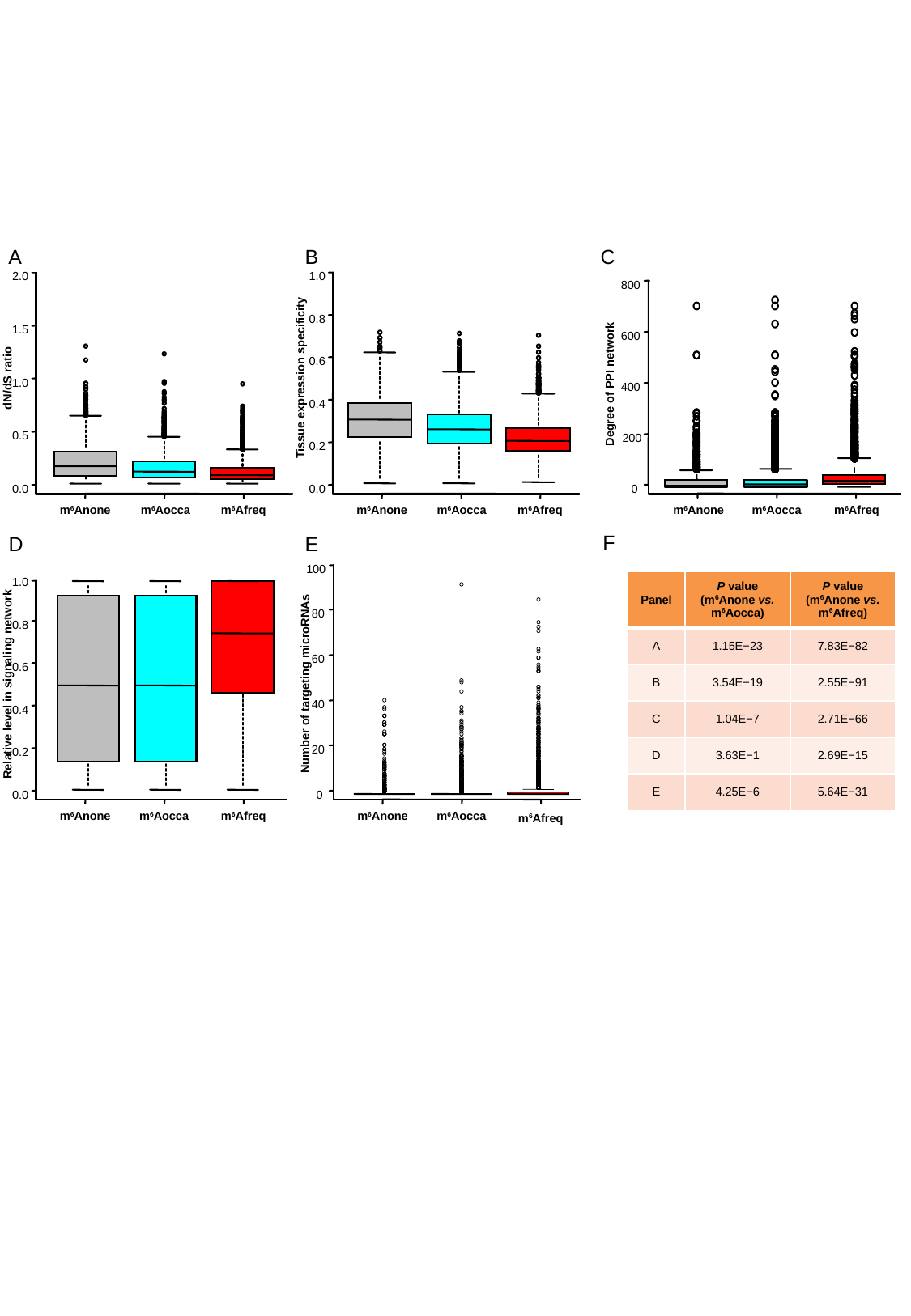

A
B
C
2.0
1.0
 800
0.8
1.5
 600
0.6
Tissue expression specificity
dN/dS ratio
1.0
Degree of PPI network
 400
0.4
0.5
200
0.2
0
0.0
0.0
m6Anone
m6Aocca
m6Afreq
m6Anone
m6Aocca
m6Afreq
m6Anone
m6Aocca
m6Afreq
F
D
E
100
| Panel | P value (m6Anone vs. m6Aocca) | P value (m6Anone vs. m6Afreq) |
| --- | --- | --- |
| A | 1.15E−23 | 7.83E−82 |
| B | 3.54E−19 | 2.55E−91 |
| C | 1.04E−7 | 2.71E−66 |
| D | 3.63E−1 | 2.69E−15 |
| E | 4.25E−6 | 5.64E−31 |
1.0
80
0.8
60
0.6
Relative level in signaling network
Number of targeting microRNAs
40
0.4
20
0.2
0.0
0
m6Anone
m6Aocca
m6Afreq
m6Anone
m6Aocca
m6Afreq
