# Supplementary material for: Comparative Analysis of Human Genes Frequently and Occasionally Regulated by m6A Modification
Source: Genomics Proteomics Bioinformatics. 2018 May 3;16(2):127–35. doi: 10.1016/j.gpb.2018.01.001 (PMC6112303; doi:10.1016/j.gpb.2018.01.001)
Supplement: Supplementary Table S1 — Information about the 38 conditions covered by the comprehensive m6A dataset. [file mmc4.docx]

**Table S1 Information about the 38 conditions covered by the comprehensive m^6^A dataset**

| **MeT-DB ID** | **Cell / tissue** | **Putative origin** | **Treatment** | **GEO accession No.** |
| --- | --- | --- | --- | --- |
| p001_p045_HEK293T | HEK293T | Kidney | No specific | GSE29714, GSE87516 |
| p002_HepG2_HGF | HepG2 | Liver | HGF (10 ng/ml) treatment | GSE37005 |
| p002_HepG2_HS | HepG2 | Liver | Heat shock (30 minutes incubation at 43ºC) | GSE37005 |
| p002_HepG2_IFN | HepG2 | Liver | IFNg (200ng/ml) treatment | GSE37005 |
| p002_HepG2_UT | HepG2 | Liver | No specific | GSE37005 |
| p002_HepG2_UV | HepG2 | Liver | UV irradiation (0.04 J/cm^2^) | GSE37005 |
| p002_Human_Brain | Brain | Brain | No specific | GSE37005 |
| p004_U2OS_CTL | U2OS | Bone | No specific | GSE48037 |
| p004_U2OS_DAA | U2OS | Bone | 100mM 3-deazadenosine treatment | GSE48037 |
| p007_HeLa_ctrl | HeLa | Uterine | Control siRNA transfection | GSE46705 |
| p009_HEK293T_siCTL | HEK293T | Kidney | Control siRNA transfection | GSE55572 |
| p009_OKMS_D0 | Fibroblast | N/A | No specific | GSE55572 |
| p009_OKMS_D5_WITH_DOX | iPSC | N/A | 5 days post OSKM induction with doxycycline | GSE55572 |
| p009_OKMS_D5_WO_DOX | iPSC | N/A | 5 days post OSKM induction without doxycycline | GSE55572 |
| p009_hESC | ESC | Embryo | No specific | GSE55572 |
| p009_hNPC | NPC | Brain | No specific | GSE55572 |
| p009_HUMAN_A549_siCTL | A549 | Lung | Control siRNA transfection | GSE55572 |
| p010_H1A_T0 | H1A | Embryo | No specific | GSE52600 |
| p010_H1A_T48_BR1 | H1A | Embryo | Differentiation towards endoderm | GSE52600 |
| p010_H1B_T0 | H1B | Embryo | No specific | GSE52600 |
| p010_H1B_T48_BR1 | H1B | Embryo | Differentiation towards endoderm | GSE52600 |
| p19_MDA_MB231 | MDA-MB-231 | Breast | No specific | GSE60213 |
| p032_A549 | A549 | Lung | No specific | GSE76367 |
| p032_H1299 | H1299 | Lung | No specific | GSE76367 |
| p035_MT4_CTL | MT4 | Lymphocyte | No specific | GSE74016 |
| p035_MT4_HIV | MT4 | Lymphocyte | HIV infection | GSE74016 |
| p041_PRIMARY_T_HIV | Primary T-cell | Lymphocyte | HIV infection | GSE54921 |
| p041_PRIMARY_T_UNINF | Primary T-cell | Lymphocyte | No specific | GSE71154 |
| p041_JURKAT_HIV | JURKAT | Lymphocyte | HIV infection | GSE85724 |
| p041_JURKAT_UNINF | JURKAT | Lymphocyte | No specific | GSE85724 |
| p045_HEK293T_ZIKV | HEK293T | Kidney | Zika virus infection | GSE87516 |
| p046_HUH7_DENV_INF | HUH7 | Liver | Dengue virus infection | GSE83438 |
| p046_HUH7_HCV_INF | HUH7 | Liver | HCV infection | GSE83438 |
| p046_HUH7_WNV_INF | HUH7 | Liver | West Nile virus infection | GSE83438 |
| p046_HUH7_YFV_INF | HUH7 | Liver | Yellow fever virus infection | GSE83438 |
| p046_HUH7_ZIKV_DAKAR_INF | HUH7 | Liver | Zika virus infection (DAKAR-41525 strain) | GSE83438 |
| p046_HUH7_ZIKV_PR_INF | HUH7 | Liver | Zika virus infection (PRVABC59 strain) | GSE83438 |
| p049_MONO_MAC6_WT | MONO-MAC-6 | Blood | No specific | GSE76414 |

*Note*: The comprehensive m^6^A modification dataset was compiled from the human m^6^A modification profiles in MeT-DB V2.0 database (http://compgenomics.utsa.edu/MeTDB/ and http://www.xjtlu.edu.cn/metdb2). The m^6^A profile of which the expression of any m^6^A methylation core components (including *METTL3*, *METTL14*, *WTAP*, *ALKBH5*, and *FTO*) was perturbed (knockout, knockdown, or overexpression) was discarded and the modification sites from the biological replicates were combined. iPSC, induced pluripotent stem cell; ESC, embryonic stem cell; NPC, neural progenitor cell; HGF, hepatocyte growth factor; IFNg, interferon-gamma; siRNA, small inference RNA; OSKM, OCT4, SOX2, KLF4, and MYC; HIV, human immunodeficiency virus; HCV, hepatitis C virus.
