# Supplementary material for: Comparative Analysis of Human Genes Frequently and Occasionally Regulated by m6A Modification
Source: Genomics Proteomics Bioinformatics. 2018 May 3;16(2):127–35. doi: 10.1016/j.gpb.2018.01.001 (PMC6112303; doi:10.1016/j.gpb.2018.01.001)
Supplement: Supplementary Table S2 — Top 20 enriched functional terms for m6Afreq genes. [file mmc5.docx]

**Table S2 Top 20 enriched functional terms for m^6^Afreq genes**

| **GO ID** | **Term** | **Corrected *P* value** |
| --- | --- | --- |
| GO:0000122 | Negative regulation of transcription from RNA polymerase II promoter | 6.10E−31 |
| GO:0016569 | Covalent chromatin modification | 5.16E−27 |
| GO:0009790 | Embryo development | 4.54E−17 |
| GO:0006396 | RNA processing | 2.75E−16 |
| GO:0032446 | Protein modification by small protein conjugation | 6.99E−16 |
| GO:0080135 | Regulation of cellular response to stress | 2.88E−13 |
| GO:0051603 | Proteolysis involved in cellular protein catabolic process | 8.57E−13 |
| GO:0051960 | Regulation of nervous system development | 3.01E−12 |
| GO:0006974 | Cellular response to DNA damage stimulus | 6.86E−12 |
| GO:0000278 | Mitotic cell cycle | 5.01E−11 |
| GO:0045936 | Negative regulation of phosphate metabolic process | 9.39E−10 |
| GO:0031329 | Regulation of cellular catabolic process | 3.07E−09 |
| GO:0007417 | Central nervous system development | 1.74E−08 |
| GO:0040007 | Growth | 8.37E−08 |
| GO:0070848 | Response to growth factor | 1.11E−07 |
| GO:1902532 | Negative regulation of intracellular signal transduction | 5.30E−07 |
| GO:0051169 | Nuclear transport | 1.52E−06 |
| GO:0033365 | Protein localization to organelle | 9.35E−06 |
| GO:0016032 | Viral process | 1.84E−05 |
| GO:0097190 | Apoptotic signaling pathway | 2.57E−05 |

*Note*: The Gene Ontology (GO) enrichment analysis was performed by gProfileR online tool (http://biit.cs.ut.ee/gprofiler). The *P* value was corrected using Benjamini−Hochberg method.
