# Supplementary material for: Comparative Analysis of Human Genes Frequently and Occasionally Regulated by m6A Modification
Source: Genomics Proteomics Bioinformatics. 2018 May 3;16(2):127–35. doi: 10.1016/j.gpb.2018.01.001 (PMC6112303; doi:10.1016/j.gpb.2018.01.001)
Supplement: Supplementary Table S3 — Top 20 enriched functional terms for m6Aocca genes. [file mmc6.docx]

**Table S3 Top 20 enriched functional terms for m^6^Aocca genes**

| **GO ID** | **Term** | **Corrected *P* value** |
| --- | --- | --- |
| GO:0045055 | Regulated exocytosis | 1.47E−09 |
| GO:0032787 | Monocarboxylic acid metabolic process | 5.17E−09 |
| GO:0051186 | Cofactor metabolic process | 7.38E−07 |
| GO:0030595 | Leukocyte chemotaxis | 9.19E−07 |
| GO:0006954 | Inflammatory response | 1.19E−06 |
| GO:0055086 | Nucleobase-containing small molecule metabolic process | 2.84E−06 |
| GO:0006614 | SRP-dependent cotranslational protein targeting to membrane | 1.82E−05 |
| GO:0003013 | Circulatory system process | 3.10E−05 |
| GO:0098754 | Detoxification | 1.34E−04 |
| GO:0019725 | Cellular homeostasis | 1.58E−04 |
| GO:0007229 | Integrin-mediated signaling pathway | 1.16E−03 |
| GO:0042445 | Hormone metabolic process | 1.54E−03 |
| GO:0015711 | Organic anion transport | 1.70E−03 |
| GO:0044282 | Small molecule catabolic process | 1.77E−03 |
| GO:0006575 | Cellular modified amino acid metabolic process | 2.35E−03 |
| GO:1901605 | Alpha-amino acid metabolic process | 2.52E−03 |
| GO:0016042 | Lipid catabolic process | 3.45E−03 |
| GO:0072521 | Purine-containing compound metabolic process | 3.60E−03 |
| GO:0050776 | Regulation of immune response | 3.65E−03 |
| GO:0098660 | Inorganic ion transmembrane transport | 4.38E−03 |

*Note*: The Gene Ontology (GO) enrichment analysis was performed by gProfileR online tool (http://biit.cs.ut.ee/gprofiler). The *P* value was corrected using Benjamini-Hochberg method.
